# Assessment of knowledge of junior doctors and non-specialists about musculoskeletal medicine

**DOI:** 10.12669/pjms.37.1.3148

**Published:** 2021

**Authors:** Saba Saif, Samina Fida, Hala Mansoor

**Affiliations:** 1 Dr. Saba Saif, FCPS. Rheumatology, FCPS Medicine. Assistant Professor, Department of Medicine, Division of Rheumatology Combined Military Hospital, Lahore, Pakistan; 2 Dr. Samina Fida, FCPS Medicine. Associate Professor, Department of Medicine, Division of Rheumatology Combined Military Hospital, Lahore, Pakistan; 3 Dr. Hala Mansoor, FCPS (Gastroentrol), FCPS Medicine, MRCP. Assistant Professor, Department of Medicine, Division of Rheumatology Combined Military Hospital, Lahore, Pakistan

**Keywords:** Musculoskeletal, Physical Examinations, Musculoskeletal Diseases, Arthritis, Undergraduate Medical Education

## Abstract

**Objectives:**

To assess the knowledge and confidence of junior doctors and non-specialists in examining and making a diagnosis of patients with musculoskeletal (MSK) diseases.

**Methods:**

This was a Cross-sectional study of 121 doctors working in medical clinics at a tertiary hospital between October and December 2019. Data were collected using a questionnaire. Doctor’s awareness regarding different MSK examination methods including gait, arms, leg, spine (GALS), pediatric gait, arms, leg, spine (pGALS) and regional examination of musculoskeletal system (REMS) was noted. Undergraduate teaching of these methods and use in their daily practice was surveyed.

**Results:**

Majority of the doctors lacked awareness about different MSK examination techniques. Awareness about GALS, REMS and pGALS was 44.6%, 59.5% and 18.2% respectively. There was significant correlation of GALS/REMS awareness with the undergraduate teaching and doctor’s clinical experience (p-value <0.05). Confidence level of doctors in diagnosing patients with adult MSK pathologies was 55%. Only few doctors were satisfied with their musculoskeletal education (29%).

**Conclusion:**

The GALS examination is a useful screening tool for junior doctors and non-specialists in a direct access setting to rule out musculoskeletal problems.

## INTRODUCTION

Musculoskeletal (MSK) conditions affect millions of people around the world. About 10 million people in the UK are suffering from arthritis.^1^ MSK disorders are ranked first in prevalence as the cause of chronic health problems, long term disabilities, and consultations with a health professional. MSK disorders are underdiagnosed in patients with medical problems. MSK conditions affect almost one in five adults.^2^ Low back pain, osteoarthritis and soft tissue rheumatism are the main diagnosis.^3^ Chronic MSK pain can cause sleep disturbance, fatigue, low mood, limited activity and participation affecting individual’s quality of life.^4^

Previously reported increases in undiagnosed cases of arthritis have highlighted the need for a simple yet sensitive screening MSK exam for identifying MSK abnormalities. Use of GALS should be considered as part of the assessment of all patients with joint problems. 5 GALS is a simple and useful screening tool for routine MSK examination in hospitals and general practice and has been integrated into the undergraduate medical curriculum. Despite this there is evidence that doctors lack awareness and, competency in MSK examination and GALS is underperformed routinely. 6 GALS provides a standardized approach to MSK examination while REMS is a more detailed examination of the relevant area, based on the ‘look, feel, move’ principle. Although being taught it is evident that GALS is under-utilized with 50% of clinicians not using GALS in their teaching.^7^ MSK examination skills can be improved by integrating peer assisted learning (PAL) into the undergraduate medical curriculum.^8^

The majority of causes of joint pains in childhood are benign, self-limiting and often trauma-related but can be the presenting features of malignancy, sepsis, vasculitis and non-accidental injury.^9^ The pGALS screen includes questions relating to pain and function but a negative response does not exclude a MSK disease, and therefore pGALS should be performed which on the average takes two minutes.^10^ To improve the confidence of junior medical officers in pediatric MSK assessment a clinical examination teaching intervention is required.^11^ Objective structured clinical examination (OSCE) based MSK workshop leads to significant improvement in knowledge and clinical skills among medical students and residents.^12^

This study was designed as joint pains are the most common presenting complaints in primary care settings, yet physicians don’t know how to examine these patients. There is no emphasis on MSK examination in undergraduate and post-graduate teaching and the majority doctors find this difficult and challenging. Most of the patients remain undiagnosed at the primary health care level being treated by quacks, Hakeem’s and other non-specialists till they develop irreversible deformities. Therefore, training the junior doctors will lead to earlier diagnosis, referral, treatment and prevention of permanent disability in such patients. The merits of introducing MSK examination into undergraduate curricula and practice should be explored. No local study was done before.

## METHODS

This study was conducted in CMH Lahore, between October and December 2019, after approval from Institutional Review Board (Ref:437/ERC/CMHLMC, Dared 07-11-19). The sample size was calculated taking frequency of adequate MSK knowledge as 13%; Confidence level 95 %, margin of error 6%. Eligibility criteria was any doctor practicing in CMH and having a daily interaction (i.e. history taking and/or clinical examination) with patients. 121 subjects selected randomly from the OPD’s and indoors voluntarily completed the survey. Subjects included mainly the junior doctors from different specialties (medicine, surgery and allied) and different non-specialists including General Practitioners and family physicians other than rheumatologists. Written informed consent was taken from each doctor and their confidentiality was maintained. Their demographic profiles (i.e. age, sex, designation, and department) and year/place of graduation were noted using a structured questionnaire (annex-111).

The subjects were asked to mark their ability to diagnose/refer common MSK pathologies (on the basis of history and examination) in both adults and school-aged children on a 10 point scale. Questionnaire also inquired about the subject’s awareness of different MSK examination methods i.e GALS, REMS and pGALS. Undergraduate teaching of these methods and use in their daily practice was also noted.

All the collected information was entered into SPSS version 22.0 and analyzed. Quantitative variables like age were presented as means with standard deviation. Qualitative variables like gender, specialty, designation, clinical experience and their confidence level in diagnosing MSK pathologies, were presented as frequency and percentages. Chi-square test of significance was applied to see significance of association of GALS, REMS, pGALS awareness with under-graduate teaching and clinical experience in years. P-value ≤0.05 was considered statistically significant.

## RESULTS

Total respondents were 121. Mean age of the subjects was 28.96+6.7. Almost half the doctors were a graduate of CMH Lahore Medical College and the rest belonged to different medical colleges all over Punjab. The study included 39 house officers, 57 residents and 25 consultants. Confidence level of doctors for diagnosing common adult and pediatric MSK pathologies was 55.4% and 33% respectively. Most of the doctors indicated that they were dissatisfied with their MSK education (71%) as there was inadequate undergraduate teaching (21.5%, 46.3% agreed to teaching for GALS, REMS) (Table-I).

**Table-I:** Demographic Characteristics.

Demographics	Frequency N (%)
Female respondents	71 (58.7 %)
Male respondents	50 (41.3%)
Time since primary medical qualification: <5 years	74 (61.2%)
Time since primary medical qualification: >5 years	47 (38.8%)
Current training specialty:	
Medicine	45 (37.2 %)
Surgery	13 (10.7 %)
General practitioner	19 (15.7 %)
Orthopedics	12 (9.9 %)
Pediatrics	13 (10.7 %)
Physiotherapists	8 (6.6 %)
Family physician	4 (3.3 %)
Other specialties	7 (5.8 %)

**Fig.1:**
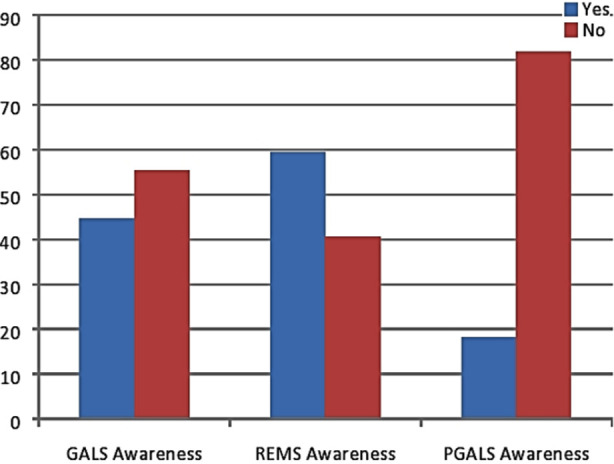
Percent Awareness of GALS/ REMS/pGALS among trainee doctors and non-specialists.

**Fig.2:**
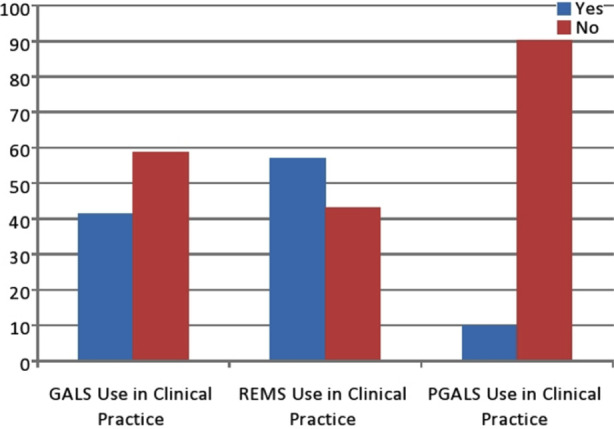
Percentage of use of GALS/REMS/pGALS among trainee doctors and non-specialist in clinical practice.

Awareness of REMS was high amongst doctors (72/121 [59.5 %]), though less were aware of GALS technique (54/121 [44.6 %]) (Fig.1). Concomitantly, more doctors reported using REMS (69/121 [57 %]) than GALS (50/121 [41.3 %]) in their clinical practice (Fig.2). Of those doctors who used GALS, most felt it had improved their ability to detect significant MSK pathology (92%).

Undergraduate exposure to pediatric MSK medicine appeared to be low in the respondents (5.8 %), and so was its awareness (18.2%). Almost 62 % of trainees reported clinical contact with children on at least a weekly basis. Of these trainees, only 16 % use pGALS in their current clinical practice, all of whom were general pediatric trainees. There was significant correlation of MSK Examinations awareness with undergraduate teaching and doctor’s clinical experience (Table-II).

**Table-II:** Comparison of GALS, REMS, PGALS with undergraduate teaching and clinical experience.

		*Undergraduate Teaching*				*p- value*

		*Yes N 26 %*		*NO N 95 %*		
GALS Awareness	Yes	26	%48	28	51.8%	0.000*
	No	0	%0	67	100%	
REMS Awareness	Yes	21	%29	51	70.8%	0.013*
	No	5	10.2%	44	89.8%	
pGALS Awareness	Yes	6	27.27%	16	72.72%	0.465
	No	20	20.2%	79	79.8%	
		*Clinical Experience*				*p-value*

		<5yrs N 74 %		>5yrs N 47 %		
GALS Awareness	Yes	23	42.59%	31	57.4%	0.000*
	No	51	76.11%	16	23.88%	
REMS Awareness	Yes	38	52.77%	34	47.22%	0.022*
	No	36	73.46%	13	26.53%	
pGALS Awareness	Yes	6	27.27%	16	72.72%	0.000*
	No	68	68.68%	31	31.31%	

* P value <0.05

## DISCUSSION

Musculoskeletal complaints constitute approximately 20% of visits to family physicians.^13^ The MSK examination is best taught in a hands-on, longitudinal fashion. Near-peer teaching refers to physicians –in –training teaching their junior colleagues.^14^ Lack of trained faculty leads to inadequate supervision of the trainees.^15^

In this survey we examined the awareness and use of structured approaches to MSK examination within the doctors in their current clinical practice. In a similar study done on trainee doctors it was found that awareness of GALS was more 91% as compared to REMS 21 % while we found out that 44.6% were aware of GALS, 59.5% of REMS and 18.18% of pGALS.^16^

The majority of doctors rated GALS technique as simple to use and easy to remember. The GALS examination is sufficiently detailed to detect MSK pathologies. Beattie KA and colleagues found out that it may be a useful tool for physiotherapists to rule out rheumatoid arthritis. The sensitivity and specificity of GALS were 71 to 86% and 69 to 93%, respectively.^17^ Another survey reported that following a very short training period, family physicians and nurse practitioners were able to use the GALS examination as a screening tool for RA.^18^

In our study most doctors were not confident in recognizing common MSK problems. In a similar study more than 30% could not confidently diagnose common rheumatic conditions, while 75% felt unable to diagnose a connective tissue disorder^19^ compared to 44.6% in our study. Similarly, 33% of our doctors could confidently diagnose pediatric MSK pathologies. Most doctors felt competent in hand assessment (68.8%) as compared to foot examination.^19^

Undergraduate teaching for MSK examination is lacking in most medical schools. The American Association of Medical Colleges claims that most medical schools do not effectively educate future physicians on MSK medicine.^20^ According to our survey 21.5%, 46.3% and 5.8% agreed to teaching for GALS, REMS and pGALS respectively. PAL is a useful adjunct to MSK clinical skills training. Trainee confidence increased from 3.7 to 89.9 (P < 0.0001) after PAL training. 21 Jandial S found that doctors lacked confidence in pediatric MSK clinical skills and many had little exposure to pGALS teaching due to time constraints and a lack of tutors.^22^

Most doctors showed dis-satisfaction with their MSK education. Al-Nammari SS found out that only 13% doctors felt they had adequate exposure to MSK medicine after completion of their foundation programmed as compared to 29% in our study.^23^ similarly in another study 80% of doctors felt that they had not received adequate teaching in MSK assessment.^19^

This study included a survey of MSK medicine practice at a local level including doctors from a broad range of specialties belonging to different medical colleges.

### Limitations of the study

First it was a single center study. Secondly a bias may have affected our result i.e. responding trainee doctors may differ from the non-responders in their interest and exposure to MSK medicine. Thirdly, junior doctor’s performance in clinical practice may not be comparable to their self-reported use and confidence in MSK examination routines.

### Strength of the study

Wider studies including multiple institutes will bring strength to our observations and thus request can be made to review the national undergraduate and postgraduate medical curriculum. The quality and quantity of exposure to MSK medicine during the foundation programme must be improved by near-peer teaching.

## CONCLUSION

Major delay occurs in diagnosing MSK diseases due to deficiencies in MSK examination skills among physicians in training and non-specialists.

### Authors Contribution:

**SS:** Conceived, designed and did manuscript writing and responsible for integrity of the article.

**SS,** **HM & SF:** Did data collection and statistical analysis.

**SS:** Did review and final approval of manuscript.
